# Botulinum Toxin for Refractory Vaginismus: A Therapeutic Evaluation of a Rare and Under-Researched Condition

**DOI:** 10.5935/1518-0557.20250051

**Published:** 2025

**Authors:** Ayse Konac, Muslum Yıldız

**Affiliations:** 1 Gelisim University, School of Health Sciences, Istanbul, Turkey; 2 AI Instructor at TalentifyLab, Dover, Delaware, USA

**Keywords:** vaginismus, penetration disorders, dyspareunia, botulinum toxin, botox

## Abstract

**Objective:**

Vaginismus is a rare and under-researched psychosexual disorder characterized by involuntary vaginal muscle contractions that impede penetration, causing significant distress. Despite its impact, large-scale studies remain scarce. This study, one of the largest of its kind, analyzes 143 patients over a six-year period (2018-2024) to assess the efficacy and safety of intravaginal Botox injections, a promising intervention that disrupts acetylcholine-mediated muscle contractions.

**Methods:**

This prospective study included 143 married women diagnosed with vaginismus between 2018 and 2024, making it one of the largest investigations on this condition. Among them, 106 patients underwent intravaginal Botox injections, while 37 patients diagnosed with vulvar vestibulitis syndrome received surgical interventions. Demographic data, including age, duration of marriage, and prior unsuccessful treatments, were recorded to assess treatment outcomes and patient characteristics.

**Results:**

This study, involving 106 women diagnosed with vaginismus, demonstrated a remarkable success rate of 81.13% (86 out of 106 patients) following vaginal Botox treatment combined with psychological support. The majority of these patients experienced significant symptom relief, enabling pain-free intercourse, often within two weeks of treatment. In contrast, 18.87% (20 patients) did not report symptom relief and continued to experience vaginismus symptoms. Participants ranged in age from 21 to 34 years, with an average age of 26.59 years, and had been married between 2 months and 8 years. The treatment was well tolerated, with no severe adverse effects reported, further supporting its safety and efficacy as a viable treatment option for vaginismus.

**Conclusions:**

This study highlights the high success rate of vaginal Botox treatment combined with psychological support, with 81.13% (86 out of 106 patients) achieving significant symptom relief and regaining pain-free intercourse. The treatment was well-tolerated and demonstrated substantial efficacy, reinforcing its potential as a viable intervention for vaginismus. However, 18.87% of patients did not experience symptom resolution, underscoring the complexity and heterogeneity of vaginismus. Notably, due to the rarity of this condition, this study represents one of the few conducted with such a large cohort, contributing valuable insights into the management of vaginismus. These findings emphasize the need for further research into individualized treatment strategies to optimize outcomes and address the varying needs of patients affected by this challenging and under-researched condition.

## INTRODUCTION

Sexual health, a fundamental component of overall well-being, is often disrupted by sexual dysfunction, which includes issues like disturbances in desire, arousal, and pain (Banaei *et al.*, 2023). Among these, vaginismus-a condition causing painful involuntary contractions of perivaginal muscles-has significant medical and psychological impacts, often resulting in unconsummated marriages and severe emotional distress for affected individuals and their partners (Badran *et al.*, 2006; Pacik, 2011; Wright & O’Connor, 2015). Despite affecting 1-7% of women globally, it remains underreported due to stigma, fear of judgment, and cultural taboos, complicating diagnosis and treatment (Reissing *et al.*, 2003; Lahaie *et al.*, 2010). Beyond its psychological and relational consequences, vaginismus also holds clinical relevance in reproductive health, as it directly affects a woman’s ability to engage in intercourse, thereby influencing marital satisfaction and natural conception.

The DSM-5 reclassified vaginismus under genito-pelvic pain/penetration disorder (GPPPD), merging it with dyspareunia, a change criticized for blurring diagnostic distinctions and potentially limiting individualized treatment approaches (Chalmers, 2024). Diagnosis relies on patient history and physical exams, but social pressures and misconceptions about sexuality often delay intervention. Traditional treatments like vaginal dilation and psychotherapy, though commonly prescribed, often provide limited or inconsistent success (Jeng, 2004; Anğın *et al.*, 2020).

Recent advances, such as botulinum toxin (Botox) injections, offer promising alternatives. Botox relaxes vaginal muscles by temporarily paralyzing them, addressing the core physical symptoms of vaginismus (Velayati *et al.*, 2019). Studies suggest it also reduces local inflammation and pain, providing relief for patients resistant to conventional therapies, particularly when integrated with psychological support (Shafik & El-Sibai, 2000; Parenti *et al.*, 2023). This study examines Botox as a treatment for vaginismus, focusing on its safety, efficacy, and integration into current therapeutic approaches.

## MATERIAL AND METHODS

The study employed a prospective clinical evaluation conducted over a six-year period (2018-2024), focusing on 143 married women diagnosed with primary vaginismus. Eligible participants had a documented history of unsuccessful treatment with non-invasive therapies and psychological interventions, ensuring a homogeneous study group of refractory cases.

To maintain diagnostic specificity, women with vulvar vestibulitis syndrome (VVS) or those undergoing surgical interventions were excluded to eliminate confounding factors. A pre-screening process involving detailed medical histories and prior treatment evaluations ensured the selection of appropriate candidates. All participants provided written informed consent after receiving a thorough explanation of the procedure, potential risks, and benefits.

### Botox Treatment Protocol

The Botox protocol was standardized yet adaptable based on individual patient response. Initial treatments employed a conservative dose to avoid excessive muscle relaxation, with subsequent doses adjusted between 100-150 units to achieve optimal symptom relief. Botulinum toxin was diluted in 2 ml of sterile saline and injected into the lateral vaginal muscles using a 1¼-inch needle, specifically targeting muscles associated with spasmodic contractions.

To enhance patient comfort and procedural tolerability, injections were administered under sedation with local anesthesia, minimizing discomfort and optimizing post-procedure recovery. This approach ensured a patient-centered treatment experience, allowing for gradual and controlled symptom relief.

To ensure diagnostic accuracy, patients with concurrent Vulvar Vestibulitis Syndrome (VVS) and vaginismus were included only if vaginismus was the primary diagnosis. This selection criterion aimed to focus on the efficacy of Botox specifically for vaginismus, minimizing potential confounding effects from VVS. Detailed clinical assessments, including history, physical examination, and pain localization tests, were conducted to differentiate primary vaginismus from secondary causes, ensuring a homogeneous study population.

### Ethical Considerations

This study was conducted in accordance with the ethical principles outlined in the Declaration of Helsinki and was approved by the clinic’s Institutional Review Board (document no. 2024-04-79, dated 15.03.2024). Patient data were anonymized, and strict confidentiality measures were implemented to protect privacy. These procedures ensured compliance with ethical standards and contributed to the reliability of insights into fertility preservation trends.

## RESULTS

The study cohort consisted of 106 women with vaginismus, with ages ranging from 21 to 34 years and an average age of 26.59 years. Participants experienced marital durations spanning from 2 months to 8 years. A significant majority, 56%, had not found relief from non-surgical methods prior to participating in the study. After undergoing vaginal Botox treatment, complemented by psychological support, 86 women reported significant improvement in symptoms, which enabled them to engage in pain-free intercourse, often within two weeks post-treatment. Conversely, 20 women did not show improvement and continued to experience symptoms. The procedure was well-tolerated with no severe adverse effects reported. The results indicate a substantial overall effectiveness of the vaginal Botox treatment in this patient population.

**Figure 1:**
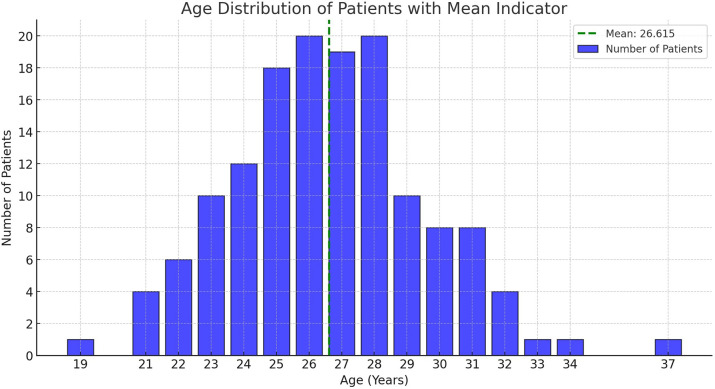
Age distribution of patients who presented with complaints of vaginismus.

Figure 1 depicts the age distribution of 143 patients who presented with complaints of vaginismus. The histogram shows a unimodal distribution, with the highest concentration of patients between the ages of 24 and 28. The peak is observed around ages 26 and 27, indicating that the majority of patients seeking medical consultation fall within this age range. The mean age of 26.615 years, represented by the green dashed line, provides insight into the central tendency of the dataset. While the age range spans from 19 to 37 years, there are fewer patients at the extremes, highlighting that vaginismus predominantly affects individuals in their mid-to-late twenties. This trend suggests that this age group is more likely to seek medical intervention, which may be influenced by social, psychological, or physiological factors.

**Figure 2:**
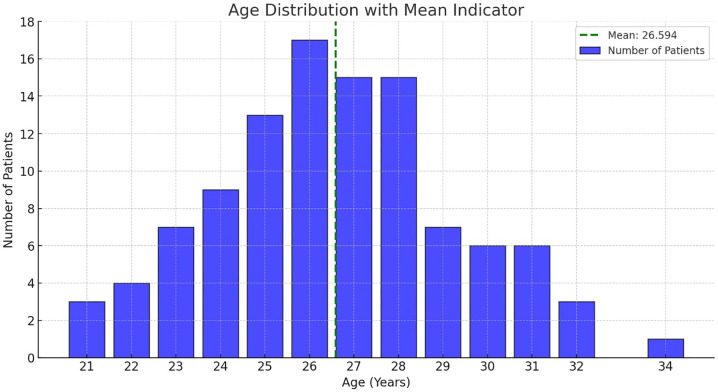
Age distribution of patients receiving Botox treatment for vaginismus.

Figure 2 illustrates the age distribution of 106 patients who underwent vaginal Botox treatment for vaginismus. The distribution is unimodal, with a peak concentration of patients between the ages of 25 and 28. The highest number of patients are observed at ages 26 and 27, suggesting that individuals in their mid-to-late twenties are more likely to seek Botox treatment for vaginismus. The mean age of 26.594 years, represented by the green dashed line, indicates the central tendency of the treated population. While the age range spans from 21 to 34 years, the lower and upper extremes have fewer cases, highlighting that Botox treatment is predominantly sought by patients in their reproductive years. This trend may reflect the clinical decision-making process or social and psychological factors influencing treatment-seeking behavior.

**Figure 3:**
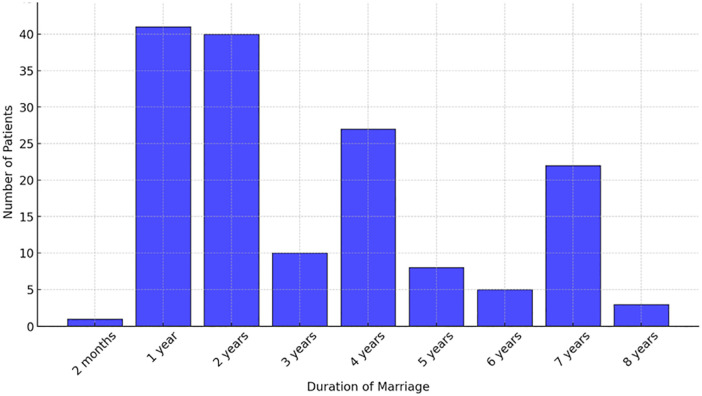
Duration of marriage of patients.

Figure 3 provides an analysis of the duration of marriage among patients presenting with vaginismus, ranging from 2 months to 8 years. The data reveal that the most common durations at which patients sought treatment were at one and two years of marriage, accounting for 41 and 40 patients, respectively. This suggests a tendency for individuals to seek medical intervention relatively early in their marriages. Additionally, significant numbers of patients also sought treatment at four years (27 patients) and seven years (22 patients) of marriage, indicating possible critical periods when symptoms may become particularly challenging. This chart thus highlights the distribution of treatment-seeking behavior over different marriage durations among patients with vaginismus, reflecting various stages at which individuals decide to seek help, possibly influenced by evolving relationship dynamics, awareness of treatment options, and the progression of symptoms.

The study cohort consisted of 106 women with vaginismus, with ages ranging from 21 to 34 years and an average age of 26.59 years. Participants experienced marital durations spanning from 2 months to 8 years. A significant majority, 56%, had not found relief from non-surgical methods prior to participating in the study. After undergoing vaginal Botox treatment, complemented by psychological support, 86 women (81.1%) reported significant improvement in symptoms, enabling them to engage in pain-free intercourse within two weeks post-treatment. Conversely, 20 women (18.9%) did not show improvement and continued to experience symptoms. The procedure was well-tolerated, with no severe adverse effects reported. These results indicate a substantial overall effectiveness of vaginal Botox treatment in this patient population.

In addition to vaginismus cases, 37 patients were diagnosed with Vulvar Vestibulitis Syndrome (VVS) and underwent surgical intervention. The diagnostic confirmation of VVS was conducted through a standardized clinical assessment, which included:

- Patient history evaluation to identify chronic vestibular pain and hypersensitivity.
- Physical examination using the Q-tip test, a validated diagnostic tool for VVS, where localized tenderness and pain were assessed by gently applying pressure to the vestibular area.
- Exclusion of other pain syndromes such as generalized vulvodynia through differential diagnosis.
- Visual and palpation-based assessment of the vestibular mucosa, checking for erythema, inflammation, and hypertrophic changes indicative of VVS.

These criteria ensured an accurate distinction between primary vaginismus and VVS, allowing for appropriate treatment allocation.

**Figure 4:**
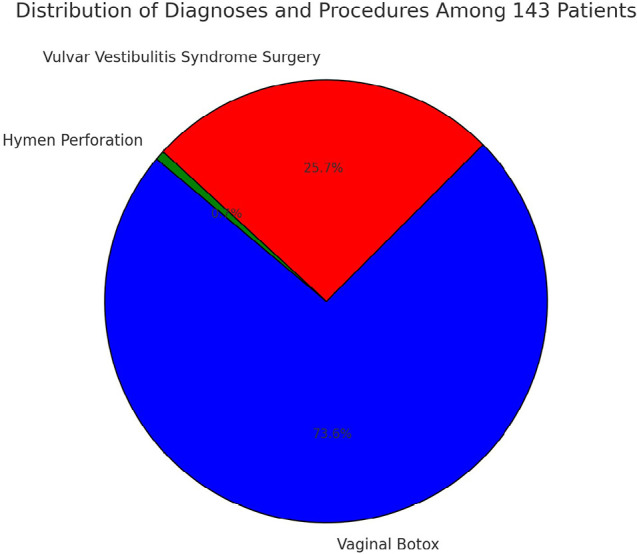
Distribution of treatment modalities.

Figure 4 displays the distribution of diagnoses and the corresponding procedures applied to a cohort of 143 married patients presenting with complaints of vaginismus over the period from 2018 to 2024. The majority of the patients, 106 out of 143 (74.1%), were treated with vaginal Botox, which represents the most commonly utilized approach within this cohort. A smaller proportion of the cohort, 37 patients (25.8%), were diagnosed with Vulvar Vestibulitis Syndrome and underwent surgical intervention. Additionally, a minor fraction (2.3%) of patients underwent hymen perforation procedures as part of their management. This distribution of treatment modalities highlights the heterogeneity of conditions presenting as vaginismus and underscores the need for precise diagnostic differentiation in clinical practice.

**Figure 5:**
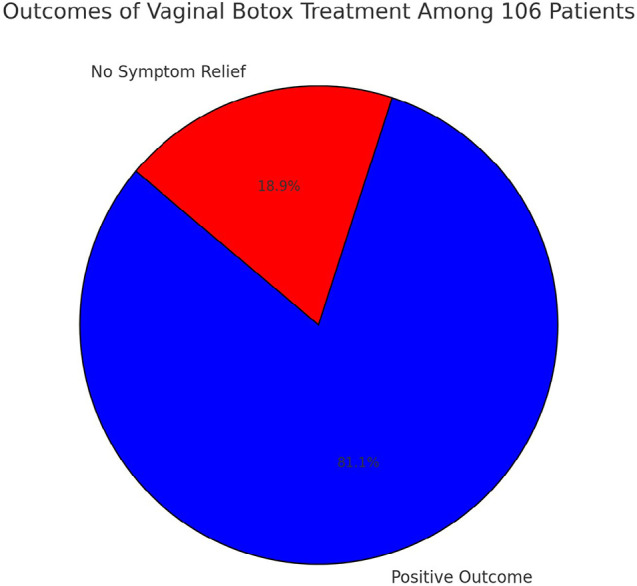
Treatment outcome.

Figure 5 illustrates the outcomes from an investigation into the effectiveness of vaginal Botox treatment, supplemented by psychological support, among a cohort of 106 patients diagnosed with vaginismus. The bar chart reveals that a significant majority of the cohort, specifically 81.13% or 86 patients, reported positive outcomes following the treatment, indicating a substantial reduction in symptoms. This notable success rate demonstrates the potential utility of vaginal Botox as an effective intervention for alleviating vaginismus symptoms. In contrast, the remaining 18.87% of the cohort, comprising 20 patients, did not report symptom relief, continuing to experience the debilitating effects of vaginismus despite undergoing the proposed treatment regimen. This variability in treatment response underscores the complexity of vaginismus as a medical condition and highlights the necessity for a deeper understanding of its underlying mechanisms to enhance therapeutic efficacy.

## DISCUSSION

In the present study, 106 women diagnosed with vaginismus, ranging in age from 21 to 34 years with an average age of 26.59 years, were treated with vaginal Botox injections accompanied by psychological support. The majority of these women, who had been married between 2 months and 8 years, sought treatment after previous non-surgical interventions had failed, a situation applicable to 56% of the participants. Post-treatment, 86 out of the 106 women reported a significant alleviation of symptoms and achieved pain-free intercourse, typically within two weeks of receiving treatment. However, six participants did not experience relief from their symptoms, continuing to struggle with vaginismus. Overall, the treatment was well-received and no severe adverse effects were reported, underscoring its safety and substantial effectiveness.

When compared to other studies, similar promising results emerge. For instance, Ghazizadeh & Nikzad (2004) observed that 75% of their 24 patients experienced satisfactory intercourse after receiving Dysport injections, with no recurrences reported during a follow-up period ranging from 2 to 24 months. Similarly, in the study by Shafik & El-Sibai (2002), all eight women treated with Botox were able to engage in intercourse, with no reinjections necessary during a 10-month follow-up. Bertolasi *et al.* (2009) reported a recovery rate of 82% in their study participants who received repeated cycles of small Dysport doses

In a randomized, controlled trial, Abbott *et al.* (2006) noted significant improvements in pelvic floor pressures and quality of life in patients receiving botulinum toxin injections compared to those who received a placebo. Additionally, Yoon *et al.* (2007) found that an initial dose of 20 units of Botox, followed by a second dose of 40 units, considerably improved pain scores in patients suffering from vulvodynia, with no recurrences over a 24-month period.

Recent studies have highlighted the efficacy of botulinum toxin (BoNTA) as a promising therapeutic intervention for vaginismus, particularly in cases refractory to conventional treatments. A prospective study by Desai *et al.* (2024) demonstrated a remarkable 95% success rate among 20 patients with severe vaginismus, all of whom had previously failed psychotherapy, vaginal dilator therapy, and pharmacological treatments. Their study employed submucosal injections of BoNTA (100-200 IU) into the bulbospongiosus, pubococcygeus, and puborectalis muscles, targeting the core muscle groups implicated in the condition. Notably, none of the participants experienced recurrence or required additional injections, and no adverse effects were reported, reinforcing the safety and durability of the treatment. These findings align with our results, where 81.13% of patients (86 out of 106) reported significant symptom relief following BoNTA treatment, further underscoring its role as a minimally invasive, well-tolerated, and effective approach for vaginismus management. While the Desai *et al.* (2024) study was limited by a small sample size and short follow-up period (four months), our study, spanning six years with a significantly larger cohort (143 patients), provides a broader perspective on long-term outcomes. Additionally, our findings reinforce the necessity of complementary psychological support alongside BoNTA, as vaginismus is a multifaceted condition influenced by both neuromuscular dysfunction and psychosocial factors. The combined evidence from both studies highlights BoNTA’s growing clinical relevance, calling for further large-scale, controlled trials to refine treatment protocols, optimize dosing strategies, and establish its role within a multimodal, patient-centered approach to vaginismus care.

The prevalence of unconsummated marriages due to vaginismus is notably high in Middle Eastern populations, where cultural and psychosocial factors often complicate treatment-seeking behaviors. A recent Egyptian study (Ramzy, 2024) demonstrated the effectiveness of a multimodal approach combining Botulinum Toxin A (BTXA) injections, gradual dilation, and psychological support, achieving a high success rate in refractory cases. These findings align with our results, where intravaginal BoNTA injections led to significant symptom relief in the majority of patients, reinforcing the importance of a combined pharmacological and rehabilitative strategy. However, while the Egyptian study reported mild adverse effects in a notable proportion of cases, our findings indicate BoNTA’s excellent tolerability with no severe complications, highlighting the need for further research to optimize treatment protocols and enhance patient outcomes.

A systematic review by Benalla (2024) underscores the potential of botulinum toxin (BoNT) injections in managing vaginismus, vulvodynia, and chronic pelvic pain, demonstrating improvements in sexual function, pain reduction, and quality of life across various studies. However, the findings highlight significant variability in dosage, administration techniques, and study methodologies, emphasizing the lack of standardized clinical guidelines for BoNT in these conditions. While the review reinforces BoNT as a promising therapeutic option, it also underscores the need for well-defined, reproducible treatment protocols to enhance its clinical application and ensure consistent patient outcomes.

These comparative results reinforce the findings of our study, highlighting botulinum toxin’s potential as a robust treatment for pelvic floor disorders like vaginismus. Notably, the high success rate and rapid onset of symptom relief observed in our study align with the outcomes seen in other research, suggesting that when combined with psychological support, Botox injections offer a highly effective treatment option for patients who have not responded to other non-surgical methods. Moreover, the consistent reporting of minimal side effects across studies emphasizes the treatment’s safety, making it a viable option for long-term management of vaginismus.

## LIMITATIONS

This study has several limitations. The lack of randomization limits the ability to establish definitive causality, though ethical concerns made a placebo-controlled trial unfeasible. The single-center design may affect the generalizability of findings, and a larger, multi-center study would provide more robust evidence. Additionally, the study primarily assessed short-term outcomes, and longer follow-up is needed to evaluate treatment durability and recurrence rates. Psychological and partner-related factors, which may influence treatment response, were not extensively analyzed and should be explored in future research. Despite these limitations, this study provides valuable insights into the efficacy of Botox in refractory vaginismus, laying the foundation for further investigation.

## CONCLUSİON

This study underscores the transformative potential of intravaginal Botox injections as a breakthrough therapeutic intervention for vaginismus, demonstrating a remarkable success rate of 81.13% (86 out of 106 patients) in achieving significant symptom relief and restoring pain-free intercourse. These findings position Botox as a highly effective and well-tolerated treatment option, offering hope to patients who have not responded to conventional therapies.

The results of this six-year study (2018-2024), one of the largest conducted on this rare condition, provide crucial insights into the real-world efficacy and safety of Botox injections for vaginismus. The vast majority of patients experienced rapid symptom relief, often within weeks, reinforcing its viability as a minimally invasive, outpatient solution. However, 18.87% of patients (20 out of 106) did not achieve symptom resolution, emphasizing the complexity and heterogeneity of vaginismus. This outcome variability signals the necessity for personalized, multidisciplinary treatment approaches that integrate pharmacological, psychological, and rehabilitative strategies to optimize outcomes for all patients.

Beyond treatment efficacy, this study provides valuable demographic insights, including age distributions and marriage durations, which are critical for shaping clinical decision-making and guiding early intervention strategies. As one of the most extensive investigations into vaginismus treatment, it fills a crucial gap in the literature, highlighting the pressing need for further high-quality clinical trials, larger patient cohorts, and extended follow-up periods to refine Botox treatment protocols and enhance patient outcomes.

While Botox represents a groundbreaking advancement in vaginismus management, its role should be viewed within a broader, holistic framework of care. As the medical community continues to grapple with the intricacies of this under-researched disorder, there remains a critical imperative to refine and expand therapeutic modalities, ensuring that all patients receive tailored, compassionate, and effective interventions that address the full spectrum of their needs.
